# Supplementary material for: Unraveling the Pathogenetic Mechanisms Underlying the Association between Specific Mitochondrial DNA Haplogroups and Parkinson’s Disease
Source: Cells. 2024 Apr 17;13(8):694. doi: 10.3390/cells13080694 (PMC11049488; doi:10.3390/cells13080694)
Supplement: Supplementary file 1 [file cells-13-00694-s001.zip › cells-2861122-supplementary figure.pdf]

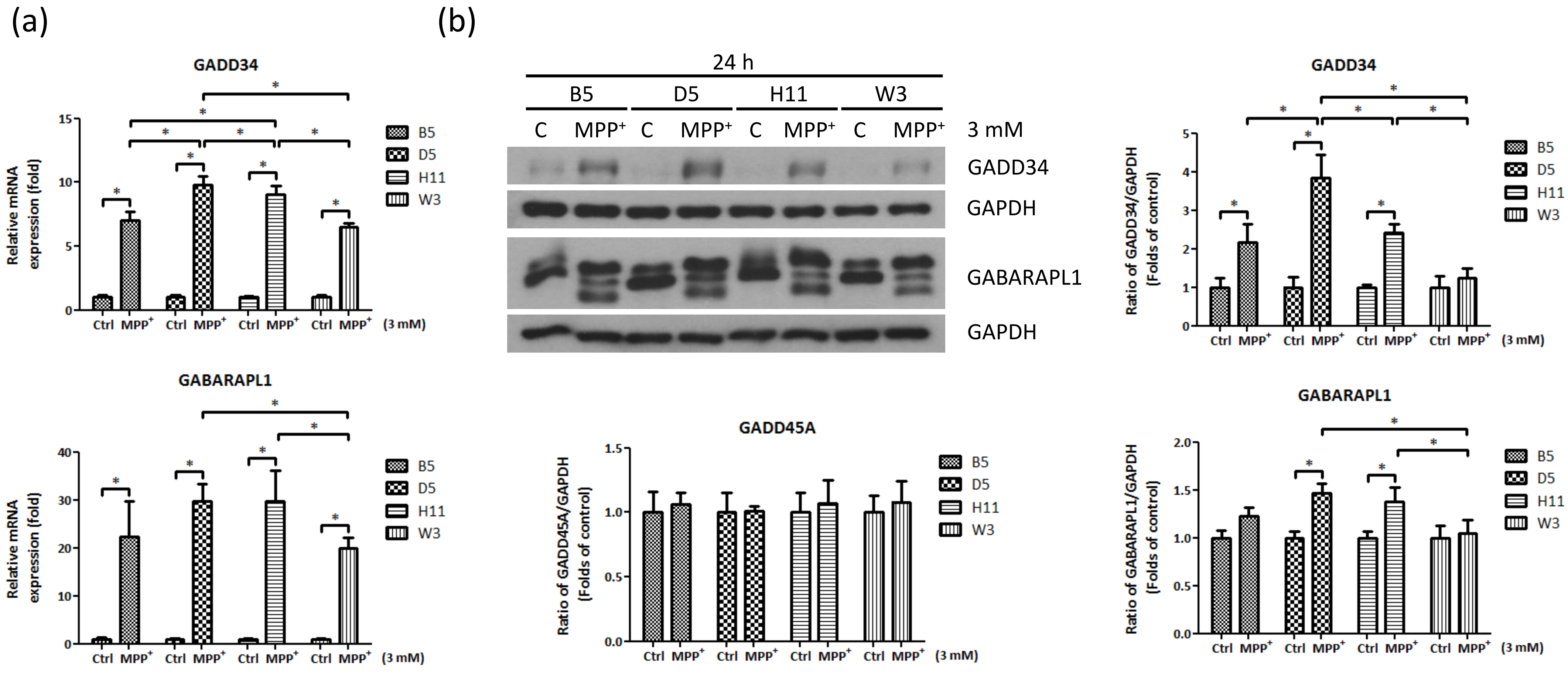

Supplemental Figure S1. Comparison of the mRNA levels (a) and protein levels (b) of GADD34 and GABARAPL1 in B5, D5, H11 and W3 cybrid cells. qRT-PCR analysis of gene expression in Cybrid cells treated with 3 mM MPP^+ for 24 h. 18S was used as internal control. Western blotting analysis of expression of GADD34 and GABARAPL1. GAPDH was as an internal loading control. Values are mean ± SD of triplicate. A *$p < 0.05$ compared to the control (one-way ANOVA with Tukey's post hoc analysis).
