# Supplementary material for: Unraveling the Pathogenetic Mechanisms Underlying the Association between Specific Mitochondrial DNA Haplogroups and Parkinson’s Disease
Source: Cells. 2024 Apr 17;13(8):694. doi: 10.3390/cells13080694 (PMC11049488; doi:10.3390/cells13080694)
Supplement: Supplementary file 1 [file cells-13-00694-s001.zip › cells-2861122-supplementary table.pdf]

cells MDPI

Supplemental tables and figures

Supplemental Table S1. For the identified DEGs from the transcriptome results of W3 cybrid.

W3 cybrid	gene_id	Ensembl Protein ID	Description	Gene type
W3-01	ENSG00000013441	ENSP00000479484	CDC like kinase 1 [Source:HGNC Symbol;Acc:HGNC:2068]	protein_coding
W3-02	ENSG00000014257	ENSP00000422036	acid phosphatase, prostate [Source:HGNC Symbol;Acc:HGNC:125]	protein_coding
W3-03	ENSG00000059728	ENSP00000443935	MAX dimerization protein 1 [Source:HGNC Symbol;Acc:HGNC:6761]	protein_coding
W3-04	ENSG00000068784	-	S1 RNA binding domain 1 [Source:HGNC Symbol;Acc:HGNC:25521]	protein_coding
W3-05	ENSG00000070669	ENSP00000175506	asparagine synthetase (glutamine-hydrolyzing) [Source:HGNC Symbol;Acc:HGNC:753]	protein_coding
W3-06	ENSG00000087074	ENSP00000469239	protein phosphatase 1 regulatory subunit 15A [Source:HGNC Symbol;Acc:HGNC:14375]	protein_coding
W3-07	ENSG00000087494	ENSP00000201015	parathyroid hormone-like hormone [Source:HGNC Symbol;Acc:HGNC:9607]	protein_coding
W3-08	ENSG00000088726	-	transmembrane protein 40 [Source:HGNC Symbol;Acc:HGNC:25620]	protein_coding
W3-09	ENSG00000095739	-	BMP and activin membrane-bound inhibitor [Source:HGNC Symbol;Acc:HGNC:30251]	protein_coding
W3-10	ENSG00000095752	ENSP00000468663	interleukin 11 [Source:HGNC Symbol;Acc:HGNC:5966]	protein_coding
W3-11	ENSG00000099860	ENSP00000468374	growth arrest and DNA damage inducible beta [Source:HGNC Symbol;Acc:HGNC:4096]	protein_coding
W3-12	ENSG00000108342	ENSP00000462991	colony stimulating factor 3 [Source:HGNC Symbol;Acc:HGNC:2438]	protein_coding
W3-13	ENSG00000108691	-	chemokine (C-C motif) ligand 2 [Source:HGNC Symbol;Acc:HGNC:10618]	protein_coding
W3-14	ENSG00000111981	ENSP00000229708	UL16 binding protein 1 [Source:HGNC Symbol;Acc:HGNC:14893]	protein_coding
W3-15	ENSG00000116717	ENSP00000482814	growth arrest and DNA damage inducible alpha [Source:HGNC Symbol;Acc:HGNC:4095]	protein_coding
W3-16	ENSG00000118523	ENSP00000356954	connective tissue growth factor [Source:HGNC Symbol;Acc:HGNC:2500]	protein_coding
W3-17	ENSG00000120217	ENSP00000370985	CD274 molecule [Source:HGNC Symbol;Acc:HGNC:17635]	protein_coding
W3-18	ENSG00000121966	-	chemokine (C-X-C motif) receptor 4 [Source:HGNC Symbol;Acc:HGNC:2561]	protein_coding
W3-19	ENSG00000125845	ENSP00000368104	bone morphogenetic protein 2 [Source:HGNC Symbol;Acc:HGNC:1069]	protein_coding
W3-20	ENSG00000126217	ENSP00000364754	MCF.2 cell line derived transforming sequence like [Source:HGNC Symbol;Acc:HGNC:14576]	protein_coding
W3-21	ENSG00000128016	ENSP00000470200	ZFP36 ring finger protein [Source:HGNC Symbol;Acc:HGNC:12862]	protein_coding
W3-22	ENSG00000130513	-	growth differentiation factor 15 [Source:HGNC Symbol;Acc:HGNC:30142]	protein_coding
W3-23	ENSG00000132002	-	DnaJ heat shock protein family (Hsp40) member B1 [Source:HGNC Symbol;Acc:HGNC:5270]	protein_coding
W3-24	ENSG00000135069	ENSP00000365773	phosphoserine aminotransferase 1 [Source:HGNC Symbol;Acc:HGNC:19129]	protein_coding
W3-25	ENSG00000136158	ENSP00000439027	sprouty RTK signaling antagonist 2 [Source:HGNC Symbol;Acc:HGNC:11270]	protein_coding
W3-26	ENSG00000136826	ENSP00000483629	Kruppel-like factor 4 (gut) [Source:HGNC Symbol;Acc:HGNC:6348]	protein_coding
W3-27	ENSG00000138166	-	dual specificity phosphatase 5 [Source:HGNC Symbol;Acc:HGNC:3071]	protein_coding
W3-28	ENSG00000139112	ENSP00000486133	GABA(A) receptor-associated protein like 1 [Source:HGNC Symbol;Acc:HGNC:4068]	protein_coding
W3-29	ENSG00000142408	ENSP00000270458	calcium channel, voltage-dependent, gamma subunit 8 [Source:HGNC Symbol;Acc:HGNC:1362	protein_coding
W3-30	ENSG00000143878	ENSP00000272233	ras homolog family member B [Source:HGNC Symbol;Acc:HGNC:668]	protein_coding
W3-31	ENSG00000146197	ENSP00000274938	signal peptide, CUB domain, EGF-like 3 [Source:HGNC Symbol;Acc:HGNC:13655]	protein_coding
W3-32	ENSG00000147027	ENSP00000275954	transmembrane protein 47 [Source:HGNC Symbol;Acc:HGNC:18515]	protein_coding
W3-33	ENSG00000148677	ENSP00000360762	ankyrin repeat domain 1 (cardiac muscle) [Source:HGNC Symbol;Acc:HGNC:15819]	protein_coding

Associated Gene Name	EntrezGene ID	locus	W3-Mppt_value	W3_value	ratio(W3-Mppt/W3)	log2ratio(W3-Mppt/W3)	p_value	q_value	significant
CLK1	1195	2:200853008-200864744	250.475	48.3028	5.185517196	2.374487888	0.00005	0.02201	yes
ACPP	55	3:132317366-132368298	0.0001	0.67618	0.00014789	-12.72319163	0.00005	0.02201	yes
MXD1	4084	2:69893559-69942945	32.015	4.68817	6.828890591	2.77165122	0.00005	0.02201	yes
SRBD1	55133	2:45388679-45612165	5.26388	20.5326	0.256366948	-1.963717819	0.00005	0.02201	yes
ASNS	440	7:97852117-97872542	152.508	32.0775	4.754360533	2.24925131	0.00005	0.02201	yes
PPP1R15A	23645	19:48872391-48876057	322.626	37.3863	8.629524719	3.109281103	0.00005	0.02201	yes
PTHLH	5744	12:27958083-27972705	23.632	2.8454	8.305334927	3.054038348	0.0001	0.0395	yes
TMEM40	55287	3:12733524-12769457	1.77935	0.0001	17793.5	14.1190627	0.00005	0.02201	yes
BAMBI	25805	10:28677341-28682939	16.3028	1.70119	9.583174131	3.260503583	0.00005	0.02201	yes
IL11	3589	19:55364388-55370463	47.2499	10.5773	4.467104081	2.159339869	0.00005	0.02201	yes
GADD45B	4616	19:2476121-2478259	124.358	15.8432	7.849298122	2.972563655	0.00005	0.02201	yes
CSF3	1440	17:40015360-40017813	17.208	146.436	0.117512087	-3.089118936	0.00005	0.02201	yes
CCL2	6347	17:34255217-34257203	10.5149	105.545	0.099624805	-3.327351201	0.00005	0.02201	yes
ULBP1	80329	6:149964006-149973710	25.1591	4.81503	5.225118016	2.385463623	0.00005	0.02201	yes
GADD45A	1647	1:67685060-67688338	274.171	61.3048	4.472259921	2.161004038	0.00005	0.02201	yes
CTGF	1490	6:131948175-132077393	581.079	137.516	4.225537392	2.079134831	0.00005	0.02201	yes
CD274	29126	9:5450502-5470566	34.488	2.77982	12.4065587	3.633031095	0.00005	0.02201	yes
CXCR4	7852	2:136114348-136118165	11.8436	1.22032	9.705323194	3.278776256	0.00005	0.02201	yes
BMP2	650	20:6767663-6780280	10.3567	1.63619	6.329766103	2.66215219	0.00005	0.02201	yes
MCF2L	23263	13:112894377-11309973	0.45865	3.94459	0.116273174	-3.10440981	0.0001	0.0395	yes
ZFP36	7538	19:39406812-39409412	35.3832	5.16418	6.851658927	2.776453336	0.00005	0.02201	yes
GDF15	9518	19:18374730-18389176	211.193	7.09293	29.77514229	4.896036497	0.00005	0.02201	yes
DNAJB1	3337	19:14514769-14565980	122.733	22.1106	5.550867005	2.472713128	0.0001	0.0395	yes
PSAT1	29968	9:78297142-78330093	301.958	61.7043	4.893629779	2.290904961	0.00005	0.02201	yes
SPRY2	10253	13:80335975-80340951	53.5249	10.0306	5.336161346	2.415802289	0.00005	0.02201	yes
KLF4	9314	9:107484851-107490482	12.1601	1.04883	11.59396661	3.535302331	0.00005	0.02201	yes
DUSP5	1847	10:110497837-11051154	286.954	52.5885	5.456592221	2.448000233	0.0001	0.0395	yes
GABARAPL1	23710	12:10212457-10223130	91.6159	13.9079	6.587328065	2.719693402	0.00005	0.02201	yes
CACNG8	59283	19:53963039-53990215	0.461264	3.69537	0.124822142	-3.002054217	0.00005	0.02201	yes
RHOB	388	2:20447073-20449445	115.842	13.2587	8.737055669	3.127147183	0.00005	0.02201	yes
SCUBE3	222663	6:35214418-35253079	2.43776	10.0934	0.241520201	-2.04978423	0.0001	0.0395	yes
TMEM47	83604	X:34627063-34657288	6.43361	1.21707	5.286146236	2.402216336	0.0001	0.0395	yes
ANKRD1	27063	10:90912095-90921276	22.3916	2.56051	8.744976587	3.128454522	0.00005	0.02201	yes

W3-34	ENSG00000148841	ENSP00000278071	inositol 1,4,5-trisphosphate receptor interacting protein [Source:HGNC Symbol;Acc:HGNC:2937(	protein_coding
W3-35	ENSG00000150347	ENSP00000308862	AT-rich interaction domain 5B [Source:HGNC Symbol;Acc:HGNC:17362]	protein_coding
W3-36	ENSG00000151012	ENSP00000424046	solute carrier family 7 (anionic amino acid transporter light chain, xc- system), member 11 [Sourc	protein_coding
W3-37	ENSG00000153714	-	leucine rich adaptor protein 1-like [Source:HGNC Symbol;Acc:HGNC:31452]	protein_coding
W3-38	ENSG00000153879	ENSP00000466022	CCAAT/enhancer binding protein (C/EBP), gamma [Source:HGNC Symbol;Acc:HGNC:1837]	protein_coding
W3-39	ENSG00000160179	-	ATP binding cassette subfamily G member 1 [Source:HGNC Symbol;Acc:HGNC:73]	protein_coding
W3-40	ENSG00000163739	ENSP00000379110	chemokine (C-X-C motif) ligand 1 (melanoma growth stimulating activity, alpha) [Source:HGNC S	protein_coding
W3-41	ENSG00000164949	ENSP00000428258	GTP binding protein overexpressed in skeletal muscle [Source:HGNC Symbol;Acc:HGNC:4234]	protein_coding
W3-42	ENSG00000166189	ENSP00000299238	Hermansky-Pudlak syndrome 6 [Source:HGNC Symbol;Acc:HGNC:18817]	protein_coding
W3-43	ENSG00000166750	ENSP00000466984	schlafen family member 5 [Source:HGNC Symbol;Acc:HGNC:28286]	protein_coding
W3-44	ENSG00000169826	ENSP00000363590	chondroitin sulfate N-acetylgalactosaminyltransferase 2 [Source:HGNC Symbol;Acc:HGNC:2429	protein_coding
W3-45	ENSG00000171388	ENSP00000391800	apelin [Source:HGNC Symbol;Acc:HGNC:16665]	protein_coding
W3-46	ENSG00000172216	ENSP00000305422	CCAAT/enhancer binding protein (C/EBP), beta [Source:HGNC Symbol;Acc:HGNC:1834]	protein_coding
W3-47	ENSG00000174343	-	cholinergic receptor, nicotinic alpha 9 [Source:HGNC Symbol;Acc:HGNC:14079]	protein_coding
W3-48	ENSG00000175868	ENSP00000433490	calcitonin-related polypeptide beta [Source:HGNC Symbol;Acc:HGNC:1438]	protein_coding
W3-49	ENSG00000176399	ENSP00000319651	DMRT-like family A1 [Source:HGNC Symbol;Acc:HGNC:13826]	protein_coding
W3-50	ENSG00000177352	ENSP00000319006	coiled-coil domain containing 71 [Source:HGNC Symbol;Acc:HGNC:25760]	protein_coding
W3-51	ENSG00000183798	ENSP00000332806	elastin microfibril interfacer 3 [Source:HGNC Symbol;Acc:HGNC:16123]	protein_coding
W3-52	ENSG00000185650	ENSP00000452119	ZFP36 ring finger protein-like 1 [Source:HGNC Symbol;Acc:HGNC:1107]	protein_coding
W3-53	ENSG00000188229	-	tubulin beta 4B class IVb [Source:HGNC Symbol;Acc:HGNC:20771]	protein_coding
W3-54	ENSG00000196668	-	long intergenic non-protein coding RNA 173 [Source:HGNC Symbol;Acc:HGNC:33791]	processed_transc
W3-55	ENSG00000198695	ENSP00000354665	mitochondrially encoded NADH:ubiquinone oxidoreductase core subunit 6 [Source:HGNC Symb	protein_coding
W3-56	ENSG00000198712	ENSP00000354876	mitochondrially encoded cytochrome c oxidase II [Source:HGNC Symbol;Acc:HGNC:7421]	protein_coding
W3-57	ENSG00000198727	ENSP00000354554	mitochondrially encoded cytochrome b [Source:HGNC Symbol;Acc:HGNC:7427]	protein_coding
W3-58	ENSG00000198763	ENSP00000355046	mitochondrially encoded NADH:ubiquinone oxidoreductase core subunit 2 [Source:HGNC Symb	protein_coding
W3-59	ENSG00000198786	ENSP00000354813	mitochondrially encoded NADH:ubiquinone oxidoreductase core subunit 5 [Source:HGNC Symb	protein_coding
W3-60	ENSG00000198804	ENSP00000354499	mitochondrially encoded cytochrome c oxidase I [Source:HGNC Symbol;Acc:HGNC:7419]	protein_coding
W3-61	ENSG00000198840	ENSP00000355206	mitochondrially encoded NADH:ubiquinone oxidoreductase core subunit 3 [Source:HGNC Symb	protein_coding
W3-62	ENSG00000198886	ENSP00000354961	mitochondrially encoded NADH:ubiquinone oxidoreductase core subunit 4 [Source:HGNC Symb	protein_coding
W3-63	ENSG00000198888	ENSP00000354687	mitochondrially encoded NADH:ubiquinone oxidoreductase core subunit 1 [Source:HGNC Symb	protein_coding
W3-64	ENSG00000198947	ENSP00000478150	dystrophin [Source:HGNC Symbol;Acc:HGNC:2928]	protein_coding
W3-65	ENSG00000204388	ENSP00000364801	heat shock protein family A (Hsp70) member 1B [Source:HGNC Symbol;Acc:HGNC:5233]	protein_coding
W3-66	ENSG00000204389	ENSP00000477378	heat shock protein family A (Hsp70) member 1A [Source:HGNC Symbol;Acc:HGNC:5232]	protein_coding
W3-67	ENSG00000209082	-	mitochondrially encoded tRNA leucine 1 (UUA/G) [Source:HGNC Symbol;Acc:HGNC:7490]	Mt_tRNA

ITPRIP	85450	10:104309697-10433840	46.712	14.0429	3.326378455	1.733952319	0.00005	0.02201	yes
ARID5B	84159	10:61901299-62096944	4.29239	0.500834	8.570484432	3.099376753	0.00005	0.02201	yes
SLC7A11	23657	4:138027421-138242349	14.2348	2.0744	6.862128808	2.778656207	0.00005	0.02201	yes
LURAP1L	286343	9:12685438-12822131	22.8089	0.9278	24.58385428	4.619639215	0.00005	0.02201	yes
CEBPG	1054	19:33373329-33382686	53.1875	11.6838	4.552243277	2.186577659	0.00005	0.02201	yes
ABCG1	9619	21:42199688-42297244	14.0804	1.57601	8.93420727	3.159339725	0.00005	0.02201	yes
CXCL1	2919	4:73869392-73871242	9.83165	58.6515	0.167628279	-2.576662543	0.00005	0.02201	yes
GEM	2669	8:94249252-94262350	124.704	12.601	9.896357432	3.306897608	0.00005	0.02201	yes
HPS6	79803	10:102065389-10206803	1.05907	7.06226	0.14996191	-2.737331987	0.0001	0.0395	yes
SLFN5	162394	17:35243035-35273655	1.67869	12.9836	0.129293108	-2.951282718	0.00005	0.02201	yes
CSGALNACT2	55454	10:43138485-43185308	51.0533	13.5229	3.775321861	1.916599645	0.00005	0.02201	yes
APLN	8862	X:129645258-129654937	4.90829	23.6466	0.20756853	-2.268340366	0.00005	0.02201	yes
CEBPB	1051	20:50184597-50192689	152.517	37.208	4.099037841	2.035285309	0.00005	0.02201	yes
CHRNA9	55584	4:40335328-40355217	1.22968	0.0001	12296.8	13.58599531	0.00005	0.02201	yes
CALCB	797	11:14904996-15082342	0.808505	0.0001	8085.05	12.98104098	0.00005	0.02201	yes
DMRTA1	63951	9:22446840-22455740	3.27959	0.693683	4.727793531	2.241167033	0.00005	0.02201	yes
CCDC71	64925	3:49162534-49166321	2.63274	15.1936	0.173279539	-2.528826787	0.0001	0.0395	yes
EMILIN3	90187	20:41340919-41366827	0.886341	4.97476	0.17816759	-2.488693171	0.0001	0.0395	yes
ZFP36L1	677	14:68787659-68796253	576.905	108.377	5.323131292	2.41227515	0.00005	0.02201	yes
TUBB4B	10383	9:137241212-137247770	27.0988	257.259	0.105336645	-3.246920677	0.00005	0.02201	yes
LINC00173	100287569	12:116533421-11653651	0.0001	2.99692	3.33676E-05	-14.87119295	0.00005	0.02201	yes
MT-ND6	4541	MT:14148-14673	87.5461	3519.1	0.024877412	-5.32901979	0.00005	0.02201	yes
MT-CO2	4513	MT:7585-8269	709.505	12082.8	0.058720247	-4.089998154	0.00005	0.02201	yes
MT-CYB	4519	MT:14746-15887	167.989	1891.96	0.088790989	-3.493442915	0.00005	0.02201	yes
MT-ND2	4536	MT:4469-5511	181.035	2894.32	0.062548371	-3.998883887	0.00005	0.02201	yes
MT-ND5	4540	MT:12336-14148	127.493	2818.88	0.045228247	-4.46663212	0.00005	0.02201	yes
MT-CO1	4512	MT:5903-7445	195.844	8219.26	0.023827449	-5.391231676	0.00005	0.02201	yes
MT-ND3	4537	MT:10058-10404	177.526	4193.48	0.042333813	-4.562045737	0.00005	0.02201	yes
MT-ND4	4538	MT:10469-12137	1387.71	10653.7	0.130256155	-2.940576548	0.00005	0.02201	yes
MT-ND1	4535	MT:3306-4262	248.143	3977.3	0.062389812	-4.002545723	0.00005	0.02201	yes
DMD	1756	X:31097676-33339441	0.0001	0.522058	0.00019155	-12.34999438	0.00005	0.02201	yes
HSPA1B	3304	6:31827734-31830255	108.725	2.10084	51.75310828	5.693573608	0.00005	0.02201	yes
HSPA1A	3303	6:31815463-31817946	87.9886	1.58853	55.38995172	5.791552376	0.00005	0.02201	yes
MT-TL1	-	MT:3229-3304	0.0001	555.957	1.7987E-07	-22.40654187	0.00005	0.02201	yes

W3-68	ENSG00000210127	-	mitochondrially encoded tRNA alanine [Source:HGNC Symbol;Acc:HGNC:7475]	Mt_tRNA
W3-69	ENSG00000210135	-	mitochondrially encoded tRNA asparagine [Source:HGNC Symbol;Acc:HGNC:7493]	Mt_tRNA
W3-70	ENSG00000210140	-	mitochondrially encoded tRNA cysteine [Source:HGNC Symbol;Acc:HGNC:7477]	Mt_tRNA
W3-71	ENSG00000210144	-	mitochondrially encoded tRNA tyrosine [Source:HGNC Symbol;Acc:HGNC:7502]	Mt_tRNA
W3-72	ENSG00000225614	ENSP00000402343	zinc finger protein 469 [Source:HGNC Symbol;Acc:HGNC:23216]	protein_coding
W3-73	ENSG00000227028	-	SLC8A1 antisense RNA 1 [Source:HGNC Symbol;Acc:HGNC:44102]	antisense
W3-74	ENSG00000227195	-	MIR663A host gene [Source:HGNC Symbol;Acc:HGNC:27662]	processed_transc
W3-75	ENSG00000233016	-	small nucleolar RNA host gene 7 [Source:HGNC Symbol;Acc:HGNC:28254]	antisense
W3-76	ENSG00000236799	-	-	lincRNA
W3-77	ENSG00000248527	-	mitochondrially encoded ATP synthase 6 pseudogene 1 [Source:HGNC Symbol;Acc:HGNC:445	unprocessed_pse
W3-78	ENSG00000251259	-	-	lincRNA
W3-79	ENSG00000255717	-	small nucleolar RNA host gene 1 [Source:HGNC Symbol;Acc:HGNC:32688]	processed_transc
W3-80	ENSG00000257556	-	-	lincRNA
W3-81	ENSG00000258479	-	long intergenic non-protein coding RNA 640 [Source:HGNC Symbol;Acc:HGNC:44291]	lincRNA
W3-82	ENSG00000261087	-	-	lincRNA
W3-83	ENSG00000261889	-	-	lincRNA
W3-84	ENSG00000263934	-	small nucleolar RNA, C/D box 3A [Source:HGNC Symbol;Acc:HGNC:33189]	snoRNA
W3-85	ENSG00000267247	-	-	lincRNA
W3-86	ENSG00000280325	-	-	TEC

MT-TA	-	MT:5586-5655	0.0001	834.454	1.19839E-07	-22.99240109	0.00005	0.02201	yes
MT-TN	-	MT:5656-5729	0.0001	1511.93	6.61406E-08	-23.84988801	0.00005	0.02201	yes
MT-TC	-	MT:5760-5891	0.0001	3972.1	2.51756E-08	-25.24339861	0.0001	0.0395	yes
MT-TY	-	MT:5760-5891	0.0001	11833.5	8.45059E-09	-26.8183016	0.00005	0.02201	yes
ZNF469	84627	16:88427470-88440757	0.37792	2.32629	0.1624561	-2.621878182	0.00005	0.02201	yes
SLC8A1-AS1	100128590	2:39786452-40611053	1.11721	0.0001	11172.1	13.44761277	0.00005	0.02201	yes
MIR663AHG	284801	20:26186919-26251526	32.0613	2.58327	12.41113008	3.633562579	0.00005	0.02201	yes
SNHG7	84973	9:136712569-136728184	446.492	56.1587	7.950540166	2.991052882	0.00005	0.02201	yes
RP11-383C6.2	102724589	10:114994656-11499659	4.52639	0.0001	45263.9	15.46607327	0.00005	0.02201	yes
MTATP6P1	-	1:585988-859446	150.204	1546.76	0.097108795	-3.364254223	0.00005	0.02201	yes
AC004069.2	-	4:105137279-105140619	0.0001	1.58873	6.29434E-05	-13.95558634	0.00005	0.02201	yes
SNHG1	-	11:62851987-62855914	429.989	115.983	3.707345042	1.890386393	0.00005	0.02201	yes
RP11-44N21.1	-	14:105093608-10509900	0.0001	3.23173	3.09432E-05	-14.98001905	0.00005	0.02201	yes
LINC00640	-	14:51333392-51385603	2.17219	0.0001	21721.9	14.40686268	0.00005	0.02201	yes
KB-1460A1.5	-	8:101166804-101169629	7.76	0.858598	9.037989839	3.176001935	0.00005	0.02201	yes
RP11-473M20.16	-	16:3156735-3157483	1.28516	0.0001	12851.6	13.64966036	0.00005	0.02201	yes
SNORD3A	780851	17:19188015-19188714	20.7783	0.0001	207783	17.6647181	0.00005	0.02201	yes
RP11-64C12.6	-	18:12230410-12231573	0.0001	1.78548	5.60073E-05	-14.12402435	0.00005	0.02201	yes
AC074183.3	-	7:84939334-84940256	46.1837	4.78677	9.64819701	3.270259366	0.00005	0.02201	yes
